# Singular Medicine

**Published:** 1860-05

**Authors:** 


### SINGULAR MEDICINE.

Isaac V. Fowler, our city Postmaster, is said to be the handsomest bachelor in New-York; but next to him is one of his "subs.," whom, meeting in Nassau st., the other day, so rosy, rubicund and round, we thus accosted:

"Why, you're as hearty as a buck!"

"Yes! I keep the bowels free, and read Hall's Journal of Health."

With the utmost confidence we advise all who are well, and desire to keep so, to follow the same prescription. As to those who are ailing, there can be no doubt that three fourths would get well of all ordinary maladies, by the same means; but the practical motto is: "Untold thousands to regain health; to retain it, not a dollar." Weeks and months of effort to arrest the progress of wasting disease; to prevent its commencement, not an hour. Such is the inconsideration, such the improvidence of nine persons out of ten, even among the educated.

